# Quantitative soil color dataset for prediction of labile and persistent organic carbon in Peruvian soils

**DOI:** 10.1016/j.dib.2026.113074

**Published:** 2026-07-11

**Authors:** Rodrigo León, Carlos Mestanza

**Affiliations:** La Molina National Agrarian University, Faculty of Agronomy, Lima 15498, Peru

**Keywords:** Soil organic carbon, Soil color, Soil carbon fractions, Pedometrics, Pedotransfer functions

## Abstract

Soil organic carbon plays a key role in climate change mitigation and exists in both labile and persistent forms. Standard quantification of both pools involves the total or partial destruction of soil samples through chemical oxidation, resulting in significant waste production and processing requirements. Given the strong association between soil organic carbon and its soil color, optical sensors offer a non-destructive and efficient alternative for its quantification. This dataset provides the analysis of labile and persistent organic carbon fractions, along with soil color data, from 329 samples randomly collected across diverse regions of Peru. Labile carbon was determined via potassium permanganate oxidation, while soil organic carbon was measured using the Walkley & Black wet combustion method. Persistent carbon was calculated as the difference between total and labile carbon. Soil colorimetric attributes were acquired with a Nix Pro 2.0 handheld spectrometer, providing comprehensive measurements in the RGB, CIELab, CIELCh, CIEXYZ, and CMYiK systems. This dataset is particularly suitable for developing pedotransfer functions that enable the preliminary estimation of labile and persistent organic carbon fractions using soil color as a rapid and cost-effective predictor.

Specifications Table

**Table:** 

Subject	Earth & Environmental Sciences
Specific subject area	Soil Science, focusing on soil organic carbon fractions and colorimetric properties to support the development of pedotransfer functions.
Type of data	Table
Data collection	A total of 329 topsoil samples (0–30 cm) were selected from the sample repository of the Soil Laboratory at the Faculty of Agronomy of the Universidad Nacional Agraria La Molina, analyzed during 2022. The samples were in the form of Air-Dried Fine Earth. Selection was restricted to mineral soil material, defined by organic carbon contents below 12%. Samples were analyzed for soil organic carbon using the Walkley & Black wet combustion method, and for labile carbon via the potassium permanganate oxidation method. Soil color parameters were acquired through direct measurement using a Nix Pro 2.0 handheld spectrometer.
Data source location	The selected samples represent 23 of Peru's administrative regions; Tumbes was excluded as no samples from this region were received during the 2022 period. Each sample is categorized by its corresponding administrative region. However, precise georeferenced coordinates are not provided to comply with the laboratory’s data protection and privacy policies regarding client confidentiality, which restrict the disclosure of detailed spatial origin.
Data accessibility	Repository name: https://figshare.com/ Data identification number: 10.6084/m9.figshare.31061614 Direct URL to data: https://doi.org/10.6084/m9.figshare.31061614 The full database is accessible on a public repository.
Related research article	None

## 1 Value of the Data

• This dataset represents a pioneering effort to establish a national-scale soil color database for Peru, covering the complex Andean Peruvian territory. Its primary value lies in providing a low-cost, non-destructive alternative for soil assessment in developing countries, where laboratory infrastructure is often limited and analytical costs are prohibitive for researchers and smallholder farmers. This alternative is achieved by linking accessible soil color attributes with high-cost variables, such as labile and persistent organic carbon.
• Consequently, these records provide a robust foundation for developing and validating pedotransfer functions aimed at estimating specific carbon fractions. As new machine learning and predictive algorithms emerge, the dataset serves as a high-quality benchmark to refine and update models, progressively enhancing prediction accuracy. Furthermore, the collection acts as a specialized reference library for the calibration of handheld sensors. Integrating these color-carbon relationships into mobile applications enables real-time, cost-effective quantification of soil organic carbon directly in the field.
• Ultimately, this dataset bridges the gap between traditional soil science and digital innovation. By promoting sustainable land management through accessible technology, it offers a scalable solution for carbon monitoring in diverse and complex environments.

## 2 Background

Monitoring soil organic carbon (SOC) and its fractions allows for the differentiation of distinct functional roles in soil health, given that SOC is a critical component for both ecosystem services and climate change mitigation [1]. Traditional quantification of SOC fractions, labile and persistent pools, relies on destructive chemical oxidation methods that are resource-intensive and generate hazardous laboratory waste. This dataset addresses the need for detailed information on labile and persistent SOC fractions by linking them with quantitative soil color measurements from various regions across Peru. This collection enables researchers to develop innovative solutions for estimating carbon pools using rapid-access variables such as soil color.

## 3 Data Description

The dataset associated with this article is hosted in the Figshare repository under the DOI: 10.6084/m9.figshare.31061614. The data is organized into a single root folder containing the following files (both are UTF-8 encoded and semicolon-delimited):

• SOC_color_dataset_v1.csv: The primary dataset containing chemical and colorimetric analysis for 329 soil samples.
• db_dictionary.csv: A structured data dictionary that defines each variable, including measurement units, data types, and the value ranges covered by each variable.

The dataset is organized in a tidy format where each row represents a unique soil sample. To facilitate the reuse of these data, Table 1 provides a summary of the variables, which are further detailed in the accompanying dictionary file.

**Table 1:** Summary of variables included in the SOC_color.dataset.csv file.

Variable Name	Description	Units
ID	Unique sample identifier	Alphanumeric
Region	Administrative region of Peru	Text
SOC	Soil Organic Carbon (Walkley & Black)	%
POXC	Labile Organic Carbon (Permanganate)	mg·kg^−1^
POC	Persistent Organic Carbon	mg·kg^−1^
L, a, b	CIELab color coordinates	Dimensionless
c, h	CIELCh color coordinates	Dimensionless
R, G, B	RGB color space values	0–255
X, Y, Z	CIEXYZ tristimulus values	Dimensionless
C, M, Yi^(*)^, K	CMYiK color model percentages	0 – 100%

(*) It is important to note that, to avoid problems with data processing, a minor change was made to the “Y” parameter between the CIEXYZ and CMYK systems, replacing the ‘Y’ with “Yi” in the CMYK system. This was done to prevent confusion regarding nomenclature and analysis.

## 4 Experimental Design, Materials and Methods

### 4.1 Data collection

**Fig. 1:**
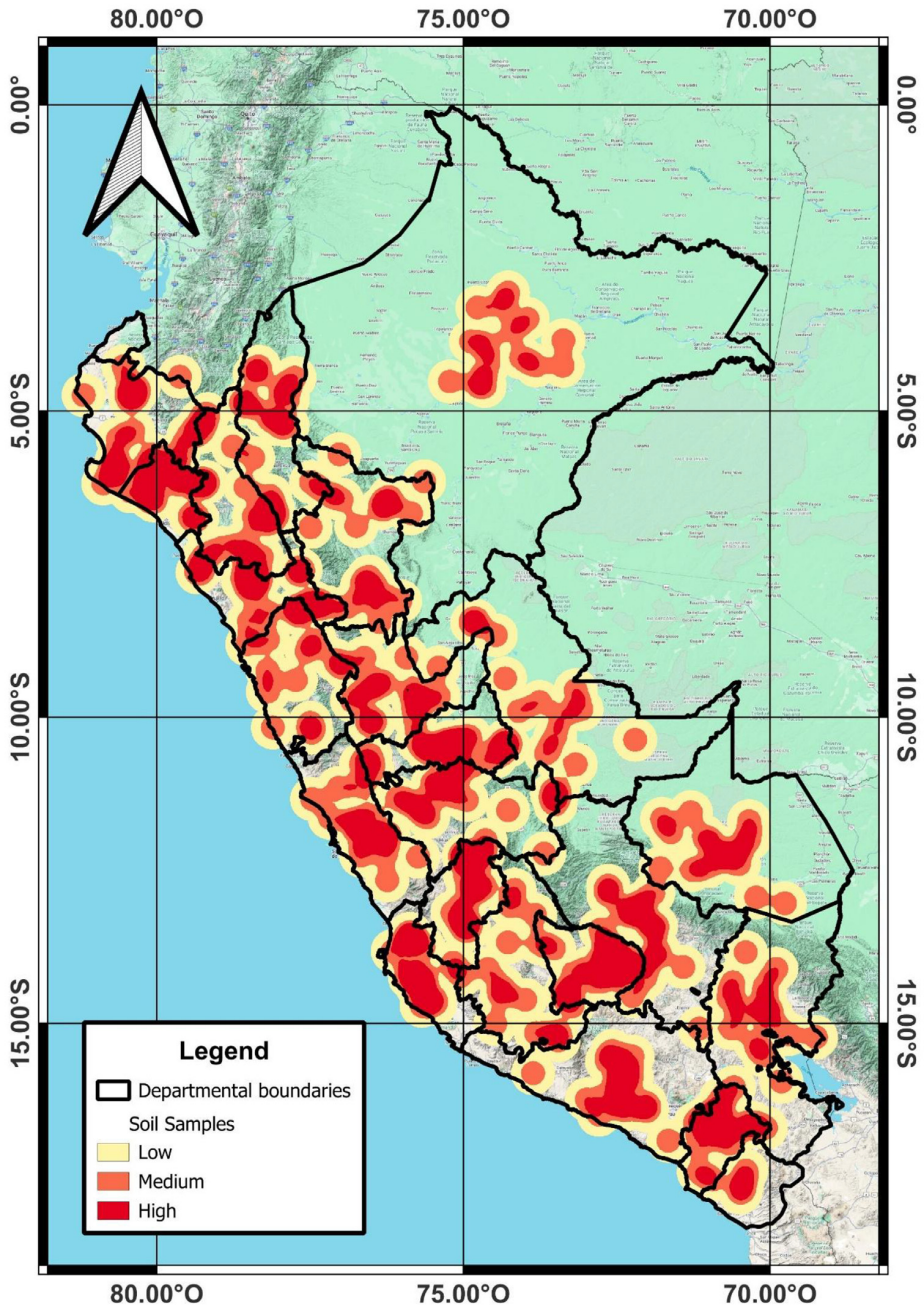
Density-based heatmap illustrating the geographic distribution of soil samples throughout the Peruvian territory.

The Soil Laboratory at the Faculty of Agronomy of the Universidad Nacional Agraria La Molina serves as a national reference, receiving approximately 30,000 samples from nearly all Peruvian regions in 2022, with the exception of Tumbes. In 2023, a representative subset was selected from this 2022 pool using a stratified sampling approach. Specifically, the createDataPartition function from the caret [2] package in R was employed, utilizing SOC percentiles as the stratification variable to ensure balanced representation across Peru’s administrative regions. The sampling design aimed to obtain 15 samples from each region; however, due to availability constraints, final counts for Huánuco, Ica, and Tacna were 14, 13, and 3 samples, respectively. Fig. 1 presents a density-based heatmap using regional centroids to verify geographical distribution, while figure confirms the nationwide representativeness of the dataset.

### 4.2 Soil analysis

Laboratory analyses were conducted to determine total SOC (%) using the Walkley & Black wet combustion method [3], while labile organic carbon (POXC) was quantified via potassium permanganate oxidation (mg.kg^−1^) [4]. Persistent organic carbon (POC) was subsequently calculated as the difference between SOC and POXC (SOC values, originally expressed as a percentage (%), were converted to mg.kg^−1^ using a multiplication factor of 10,000). Any negative POC values resulting from this subtraction were excluded from the final dataset. Soil color was measured using a Nix Pro 2 handheld spectrometer (Pro Color Sensor™, Nix Sensor Ltd., Ontario, Canada). For colorimetric characterization, soil samples were uniformly spread into 60×15 mm glass Petri dishes. The spectrometer was placed directly on the soil surface to minimize the distance between the sensor aperture and the sample, ensuring measurement consistency. Data were recorded across the RGB, CIEXYZ, CIELab, CIELCh and CMYiK color systems.

## Limitations

To ensure the appropriate application of these records, certain limitations should be noted. First, in compliance with the Peruvian Law No 29,733 on Personal Data Protection [5] regarding laboratory client confidentiality, precise georeferenced coordinates are unavailable; samples are identified only by their administrative regions of origin. This restricts the dataset's use for spatial modeling. Second, the collection is limited to topsoil (0–30 cm), thus not representing vertical carbon dynamics. Third, geographical gaps exist as Tumbes region is not represented. Finally, a potential selection bias exists because the samples originate from a laboratory repository of samples submitted by third parties. Consequently, the dataset may be skewed toward managed agricultural soils rather than natural ecosystems or unmanaged lands. Researchers should account for these factors when integrating these data into broader national or global soil models, as the variability captured reflects primarily the areas and land uses serviced by the laboratory during the 2022 period.

## Ethics Statement

The authors confirm that they have read and followed the ethical requirements for publication in Data in Brief. This work does not involve human subjects, animal experiments, or any data collected from social media platforms.

## CRediT Author Statement

**R. León**: Conceptualization, Software, Formal analysis, Investigation, Writing - Original Draft.

**C. Mestanza:** Conceptualization, Methodology, Validation, Resources, Writing - Review & Editing, Supervision.

## Declaration of Generative AI and AI-assisted Technologies in the Manuscript Preparation Process

During the preparation of this work the author used Gemini (Google) in order to translate technical sections from Spanish to English and review the grammar and structure of the text. After using this tool, the author reviewed and edited the content as needed and takes full responsibility for the content of the published article.

## Data Availability

FigsharePeruvian Quantitative Soil Color and Carbon Fractions Dataset (Original data)
